# Should taurine be pursued further as a supportive nutraceutical for Duchenne muscular dystrophy? Gathering evidence in a zebrafish disease model to confirm or refute the benefits - Stage 1 Report

**DOI:** 10.3389/fnut.2026.1903256

**Published:** 2026-08-28

**Authors:** Lenie Vanhove, Marthe Dias, Delphine De Coninck, Charlotte Grootaert, Andreja Rajkovic, Delfien Syx, Arnaud Vanlander, Jan L. De Bleecker, Boel De Paepe

**Affiliations:** 1 Mitochondrial Investigations Laboratory, Department of Pediatrics, Division of Child Neurology & Metabolism, Ghent University, Ghent, Belgium; 2 Laboratory for Neuropathology, Department of Neurology, Ghent University, Ghent, Belgium; 3 Laboratory of Food Chemistry and Human Nutrition, Department of Food Technology, Safety and Health, Ghent University, Ghent, Belgium; 4 Center for Medical Genetics, Ghent University Hospital, Ghent, Belgium; 5 Neuromuscular Reference Center, Ghent University Hospital, Ghent, Belgium

**Keywords:** Duchenne muscular dystrophy, muscular dystrophy, nutraceuticals, supportive treatment, taurine

## Abstract

Duchenne muscular dystrophy (DMD) is an X-linked neuromuscular disorder characterized by progressive muscle degeneration resulting in severe muscle weakness and motor function loss. Historically, therapeutic innovation has been facilitated by research in the mdx mouse, which is the most commonly used model for studying DMD. However, the inherent limitations of this model necessitate the formulation of alternative strategies. The present study proposes the zebrafish as an additional pre-clinical disease model in which to evaluate the benefits of nutraceuticals for DMD. The focus of this study will be taurine, a non-protein building amino acid that has emerged as a promising disease-modifying supplement for muscle wasting conditions, including aging-associated sarcopenia and DMD. The present study will evaluate the potential of early taurine supplementation to enhance the efficiency of autophagy and to improve mitochondrial health in a zebrafish DMD model. The study will facilitate a more rigorous examination of the claims pertaining to the beneficial effects of taurine on DMD progression, thereby informing the decision-making process regarding its potential implementation in human disease management.

## 1 Introduction

The rare condition of Duchenne muscular dystrophy (DMD), a severe progressive X-linked genetic disorder (1), and the prevalent condition of aging sarcopenia have in common the fact that patients suffer from reduced skeletal muscle quantity, strength and performance (2). DMD is caused by the absence of dystrophin protein, leading to fragility of muscle cells. Patients lose ambulation around the age of 12 years, and face an early death usually due to heart or respiratory failure. The therapeutic approach currently includes molecular therapies that aim to (partially) restore dystrophin expression, however, the disease remains uncurable to date. Therapeutic follow-up for DMD emphasizes the management of symptoms by reducing inflammation with steroids, moderating muscle tissue loss through physical therapy, and providing cardiac and respiratory support. Consequently, the quest to identify supportive therapies that enhance patients' quality of life remains worthwhile.

Dietary supplementation is regarded as a soft therapeutic intervention, yet it is one that possesses disease-modifying potential. For DMD, nutraceuticals may be recommended to support muscle function and improve energy metabolism. The use of creatine monohydrate is the most widely supported intervention to which the scientific community ascribes muscle strengthening properties (3). Vitamin D (4) and calcium have been shown to counteract bone weakness resulting from corticosteroid use (5). In addition, coenzyme Q10 and other antioxidants have been proposed to support muscle function (6). Taurine (2-aminoethanesulfonic acid), a naturally occurring non-protein building amino acid, has also surfaced as a promising supportive treatment for DMD, based upon its anti-inflammatory and muscle damage repairing properties (7). Preclinical studies on the disease's mouse model mdx showed taurine supplementation to improve muscle strength, to reduce fatigue, and to mitigate oxidative stress (8). However, the benefits are slight and unwanted effects have also arisen, most particularly lower body weight and growth retardation (9, 10). Further exploratory studies are therefore warranted to ascertain whether or not taurine formulae are both safe and effective for DMD. In addition, enhancement of our knowledge of the cellular pathways underlying taurine-induced effects are essential in order to fully address suitability of taurine supplements for DMD.

An important shortcoming of available preclinical studies is that almost the entirety of the available scientific evidence has been generated in the mdx mouse. The disease phenotype in this murine model, albeit the standard for studying DMD, differs substantially from the human condition. We therefore propose to investigate a zebrafish model of DMD that has a better fit with human disease characteristics (11). The dmd^ta222a/ta222a^ strain carries an autosomal recessive nonsense mutation in exon 4 of the zebrafish *dmd* ortholog, resulting in the lack of dystrophin protein (12). The model displays progressive muscle degeneration, fibrosis and inflammation, and fish do not survive to sexual maturity. In addition, zebrafish have several inherent advantages for translational research. Fish larvae develop externally and are optically transparent, allowing non-invasive visualization of skeletal muscle pathology. Drug testing is simplified by direct absorption of small molecules through the skin, eliminating the need for invasive administration, or differences in nutritional uptake. Investigations in zebrafish can accelerate the development of nutraceuticals for treating humans. Zebrafish models are convenient to determine if aging-associated decline in muscle strength can be prevented by stimulants of mitochondrial health (13), indicating they are an excellent complementary pre-clinical tool. To our knowledge, only one study of taurine administration in animal models included an experiment with *dmd*^−/−^ zebrafish to date (14). The study is a combination of rat and zebrafish model experiments, using the dmdPC2/PC2 zebrafish strain (RRID:_ZFIN_ZDB-GENO-131206-1) with a C>T point mutation in exon 32 causing a premature stop. In this study, zebrafish were treated with 1 mM taurine in E3 medium from 2 to 6 dpf, at which point movement was recorded and analyzed after *post hoc* genotyping. Distance traveled was significantly reduced in *dmd* deficient fish, but unaltered by taurine treatment. Taurine treatment significantly increased taurine content in wildtype and heterozygous larvae, but not in *dmd* deficient larvae, the latter had significantly higher taurine levels than wildtype irrespective of treatment. The authors recorded no other experiments in this zebrafish model and more studies are to be welcomed to determine if zebrafish represent an applicable disease model for therapeutic discovery for DMD.

Nutraceuticals are at best able to modify DMD disease and do not have a direct effect on dystrophin expression, hence outcome measures relevant to secondary disease characteristics need to be chosen that are quantifiable and meaningful. We propose to quantify autophagic and mitochondrial health as objective evaluators of potential therapeutic benefit, since impaired autophagic clearance of cellular components (15) and mitochondrial dysfunction (16) have consistently been described in DMD. The cell's autophagic machinery needs to function in a coordinated manner, engulfing unwanted cytoplasmic cargo and transporting it to lysosomes for elimination. Due to its destructive/reconstructive nature, autophagic processes need to be meticulously finetuned to the requirements for maintaining cellular homeostasis. This complex and multilayered system is primarily governed through the activities of nutrient-sensing kinases. Mechanistic target of rapamycin (mTOR), a member of the PI3K-related kinase protein family, plays its role as a regulator via the formation of mTORC1 and mTORC2 protein complexes (17) and associations with autophagy-related gene (ATG) proteins. The degradation of unwanted cellular components is facilitated by the autophagy cargo receptor sequestosome 1 (SQSTM1) and activation of the autophagosome membrane marker microtubule-associated protein 1A/1B-light chain 3 (LC3). DMD muscle displays defective clearance of damaged mitochondria through mitophagy, a process involving phosphatase and tensin homolog-induced putative protein kinase 1 (PINK1) and the E3 ubiquitin ligase Parkin, which leads to the accumulation of dysfunctional mitochondria. The mitochondrial process of oxidative phosphorylation, carried out by five mitochondrial protein complexes working together to produce ATP, becomes impaired, and damaging reactive oxygen species (ROS) accumulate.

We developed a strategy to help confirm or refute the claims of benefits associated with taurine supplementation in DMD. Primary endpoints will be survival in response to treatment with 1, 1.5 and 2 mM taurine in the swimming water from 0 to 20 dpf, and energy metabolism in 5 dpf zebrafish larvae. With these experiment we aim to determine if the treatment regimen has a positive effect upon survival and mitochondrial health of *dmd*^−/−^ larvae, generating dichotome answers of benefit yes or no. Zebrafish have been studied to understand the aging-associated skeletal muscle decay process. There is evidence that mitochondrial decline is a determinant of muscle damage, and this process can be delayed by resveratrol, a stimulant of mitochondrial activity (13). The study will carry on with investigating secondary endpoints that will provide mechanistic evidence whether enhanced mitochondrial activity, restorative autophagy and oxidative stress response are involved using transcriptomics and proteomics. This to verify if, in the absence of observable functional effects, hidden benefits can be picked up in biomarkers know to be relevant to disease progression. We speculate that a single bioactive compound might not be powerful enough to substantially ameliorate disease progression. In evidence, the multi-compound Cordyceps militaries mushroom supplement provokes multiphase health benefits related to accessory and/or synergistic effects powerful enough to improve mitochondrial respiration and reduce oxidative stress (18, 19). The zebrafish model would allow pre-clinical testing of taurine for later integration into a combinatory anti-inflammatory, molecular and nutraceutical therapeutic scheme. This study aims to generate data to inform such further pursuit of taurine as a nutraceutical intervention for DMD and provide valuable insight into its mechanisms of action.

## 2 Methods

### 2.1 Animal model

Zebrafish from the dmd^ta222a/ta222a^ strain (RRID:_ZFIN_ZDB-GENO-110429-6) will be supplied by the European Zebrafish Resource Center (EZRC; Karlsruhe Institute of technology, Germany). The strain contains a point mutation T>A on chromosome 1, leading to a premature stop. It represents a recessive disease model, *dmd*^−/−^ individuals do not reach maturity, therefore breeding will be conducted between *dmd*^+/−^ heterozygotes theoretically generating 25% *dmd*^−/−^ offspring. Collected eggs will first be kept in Petri dishes and placed in an incubator at 28°C, until 5 dpf in E3 medium (17.2 g NaCl, 0.76 g KCl, 2.9 g CaCl_2_·2H_2_O, 4.9 g MgSO_4_·2H_2_O per liter). From 5 dpf zebrafish will be kept in a semi-closed recirculating housing systems (ZebTech, Techniplast, Buguggiate, Italy) at 27°C, pH 7.5, conductivity ±550 μS and a 14/10 light-dark cycle. From 5 dpf to 20 dpf, zebrafish will be fed with Zebrafeed < 100 (Sparos, Olhao, Portugal) and rotifers (in-house culture). From 20 dpf zebrafish will be fed with Zebrafeed 100–200 (Sparos) supplemented with Gemma Micro dry food 300 (Inve, Baasrode, Belgium) and Micro Artemia (Ocean Nutrition, Essen, Belgium). Zebrafish line maintenance will adhere to standard protocols for animal wellbeing, minimalizing pain, distress and discomfort as much as possible. Daily monitoring of parameters for water quality, nutrition, limitation of pathogens and rearing density will maintain optimal living conditions. Fish that fail to meet standards for natural behavior, swimming capacities and healthy morphology will be euthanized to alleviate suffering. All experimental procedures have already been approved by the Animal Ethics Committee of Ghent University, permit no. ECD 24/26, valid from 04/07/2024 until 03/07/2028.

### 2.2 Treatment

Dosage of taurine (T8691, Merck, Hoeilaart, Belgium) has been determined based upon experiments in mdx (20) and preliminary experiments in zebrafish, set to the range between 1 and 2mM in E3 medium with treatment starting approximately 3h after fertilization. Embryos and larvae younger than 5 dpf, a stage at which pain perception has not yet developed, are euthanized by bleaching for a minimum of 10 min in 5% sodium hypochlorite. Larvae 5 dpf or older are euthanized by submerging in 1 g/L ethyl 3-aminobenzoate methanesulfonate (tricaine, MS-222, Merck Life Sciences, Hoeilaart, Belgium) for a minimum of 10 min, after which death is confirmed by the absence of opercular movement, as observed under a microscope.

### 2.3 Phenotyping using birefringence

Muscle integrity will be assessed in 4 dpf zebrafish larvae using birefringence based upon a published procedure (21) under a Zeiss Axio Observer 21 microscope operated with ZEN software version 3.10 (Carl Zeiss, Oberkochen, Germany). Unanesthetized larvae will be placed in a small drop of E3 medium on a glass-bottom Petri dish that will be positioned between two glass-polarizing filters, rotating the top polarizing filter to maximize the visualization of light refracting through the striated trunk muscle. Larvae displaying bright, well-organized myotomes will be classified as normal, while those with a patchy, disorganized arrangement of sarcomeres will be classified as abnormal.

### 2.4 Genotyping using Sanger sequencing

DNA will be extracted by adding 50 μL of 50 mM sodium hydroxide (NaOH) at 95 °C for 20 min. Samples will be protected from degradation by adding 5 μL of 1 M Tris–HCl (pH 7.0) and kept frozen at −20 °C. Polymerase chain reaction (PCR) amplification will be carried out using the *dmd*-specific primers: Forward: 5′-CTGGTTACATTCTGAGAGACTTTC-3′, Reverse: 5′-AGCCAGCTGAACCAATTAACTCAC-3′ (IDT, Integrated DNA Technologies, Coralville, Iowa, USA) in PCR master mix (10 μL KAPA2G Robust HotStart ReadyMix 2x (Hoffman-La Roche, Bazel, Switzerland) with the following thermocycling conditions: 95 °C for 3 min, 35 cycles of 95 °C for 15 sec, annealing at 52 °C for 10 sec and 72 °C for 15 sec, and extension at 72 °C for 1 min. PCR products will be purified by adding an enzyme mix of Antartic Phosphatase and Exonuclease I (New England Biolabs, Ipswich, MA, USA) incubated at 37 °C for 15 min and heat inactivated at 80 °C for 20 min. The sequencing reaction will be carried out with the forward primer and fluorochrome-labeled dideoxynucleotides (BigDye Terminator v3.x Cycle Sequencing kit; Applied Biosystems, Merck Life Science, Hoeilaart, Belgium) in ABI-buffer (200 mM Tris-HCL pH 8, 8; 5 mM MgCl2) with 5% dimethylsulfoxide. Thermocycling conditions will be the following: 95 °C for 5 min, 25 cycles of 95 °C for 10 sec, 55 °C for 5 sec, 60 °C for 2 min and 15 °C for 10 min. Purification will be performed using magnetic CleanDTR beads (GC Biotech, Waddinxveen, Netherlands), two washes with 85% ethanol and elution with millipure water. Sequencing will be performed on an ABI PRISM 3730XL Genetic Analyzer (ABI 3730, Applied Biosystems) and analyzed using MEGA12 software (22).

### 2.5 Survival assay

Zebrafish will be kept in E3 medium with or without 1, 1.5 and 2 mM taurine, supplemented with Zebrafeed < 100 (Sparos) and rotifers (in-house culture). Each weekday, half of the volume will be replaced by fresh medium. Monitoring will be initiated at 5 dpf and continued until 20 dpf. Mortality will be recorded daily (excluding weekends), collecting the bodies to enable retrospective genotyping. At the end of the survival, all remaining fish will be euthanized. For survival analysis, the Kaplan-Meier estimate will be used, plotting survival over time in a step-function curve and using non-parametric statistical analysis to test probability (23).

### 2.6 Real-time live cell metabolism analysis

With Seahorse Extracellular Flux Analysis (Agilent, Santa Clara, CA, USA), the two major energy-producing pathways of cells, i.e., mitochondrial respiratory capacity quantified as oxygen consumption rate (OCR) and glycolysis and subsequent lactate production quantified through extracellular acidification rate (ECAR), will be measured in individual zebrafish larvae. The day before the experiment, 4 dpf larvae will be phenotyped using methodologies described under section 2.3. At 5 dpf, larvae will be selected that meet the standards of normal general morphology and escape reflexes determined as a preserved touch-evoke response. Energy metabolism profiles will be determined using the XF96 Seahorse Efflux Analyzer (Agilent) based upon a published protocol (24), measuring OCR in pMol/min and ECAR in mpH/min. Measurements will be done every 5 min: 7 times under basal conditions, 6 times with 10 μM oligomycine, 7 times with 2.5μM FCCP, and 3 times with 1 μM rotenone/antimycine A mixture. Measurements negative or nearing 0 will be excluded from the analysis, as will flatliners which are presumed to represent larvae that died from the start of the assay. Results will be analyzed with Wave Desktop Software version 2.6 (Agilent).

### 2.7 Quantitative transcriptomics

Samples will be prepared from 20 pooled fish to obtain sufficient content for performing sets of high-performance real-time quantitative PCR (RT-qPCR) reactions. Total RNA will be extracted with TRIzol (Thermo Fisher Scientific) and purified using the RNeasy mini kit (Qiagen, Venlo, The Netherlands) according to the manufacturer's specifications. The cDNA, prepared using the iScript cDNA synthesis kit (Bio-Rad, Hercules, CA, USA), will be amplified by 35 cycles of 5 min 25 °C 30 min 42 °C 5 min 85 °C in SsoAdvanced Universal SYBR Green Supermix (Bio-Rad), with primer sets for amplifying *hmox1a* (CCACGTCAGAGCTGAAAACA, AGCGCTCGGTAGATCTCGTA), *sod1* (GGGCCAACCGATAGTGTGAG, ACCCTTCCCCAAGTCATCCT), *sod2* (ACTGTGTGACGGACTAGAGC, CAGATGTGAGGCTCAAGTGC), *map1lc3b* (GACATTTGAGCAGCGGGTGGA, GGTTGGAGTTGAGTTGGAGGC), *sqstm1* (CCCCCTTGGCATAGATGTGG, TTGTTCCCTCACTGACGCTC), *cox4* (CAAGTTTGTGCAGCAGCTG, CAAAGAAGAAGATTCCTGCAAC), *cox5ab* (GGTCACCGGAGCTTCAGGAT, TCGAGCCGAGAGGTAGAAAAACC), *atp5fa1* (GTCTGTCTGTGTCCCGTGTC, AGCAGCAACCTCACGATACT), *cs* (ATCCGTTTCCGTGGTTACAG, AGACAGCCAACTGACCTGCT), *mtor* (GCCGCTTTGCCAACTATTT, TCGTCTGCCTTCATTCCTG), *pink1* (CTGATGACGTTCAGCTGGTG, CCACAGACTGATGTGCAGGA), and the housekeeping gene *rns18* (TTTGCCATCACCGCTATTAAG, CTTTCCTCAGAACCACATGG). Melting curve analysis will be conducted using qBase+ software (Biogazelle, Ghent, Belgium).

### 2.8 Quantitative proteomics

Material from 10 fish will be pooled per genotype or phenotype to prepare protein samples with sufficient protein content for performing sets of Western blots, by homogenizing the tissues in 50 μL of extraction buffer: 50 mM Tris, 2 mM EDTA, pH 7.4, cOmplete, EDTA-free Protease Inhibitor Cocktail (Sigma Aldrich, Saint Louis, MO, USA) using a disposable pellet pestle. The homogenate will be centrifuged at 13,000 rpm for 15 min and the supernatant, containing cellular proteins, collected. Sample protein concentration will be estimated based upon absorption at 280 nm using a BioDrop μLite+ microvolume spectrophotometer (Harvard Bioscience, Holliston, MA, USA) and transferred into cryovials to be stored at −80 °C until used. Samples will be dissolved in Laemmli buffer with 10% beta-mercaptoethanol (Bio-Rad), boiled for 2 min, and subjected to polyacrylamide gel electrophoresis using the Bio-Rad stain-free system following the manufacturers' specifications (Bio-Rad). Per lane, samples representing approximately 30 μg of protein will be loaded onto 4%−20% Mini-PROTEAN TGX stain-free gels (Bio-Rad), alongside molecular weight markers Precision Plus Protein All Blue (#1610373, Bio-Rad) and Precision Plus Protein Unstained (#1610363, Bio-Rad). Blotting on low-fluorescence PVDF membranes will be performed using the Trans-Blot Turbo™ Transfer System (Bio-Rad). A stain-free blot image will be acquired using the ChemiDoc Imager to visualize total protein per lane, which will be used for normalization purposes. The membrane will then be sequentially incubated with rabbit anti-mTOR (#2972, Cell Signaling, RRID:_AB_330978) and anti-LC3II (#3868, Cell Signaling, RRID:_AB_2137707) in milk blocking buffer. Horse anti-rabbit secondary antibody conjugated to horseradish peroxidase (HRP) (#7074, Cell Signaling, RRID:_AB_2099233) and Pierce ECL substrate will detect proteins of interest via chemiluminescence using the ChemiDoc Imager (Bio-Rad). An additional staining to evaluate protein load will be done using the Western Breeze Chromogenic Immunodetection System (Thermo Fisher Scientific), either with mouse monoclonal mitochondrial marker anti-ATP5A (15H4C4, Abcam, Cambridge, UK), or general cell marker anti-glyceraldehyde-3-phosphate dehydrogenase (GAPDH) (#60004-1-Ig, Proteintech, RRID:_AB_2107436). Quantitative analysis will be performed using Image Lab software (Bio-Rad), with normalization to total protein quantities derived from the stain-free blot, or to housekeeping protein GAPDH.

### 2.9 Power calculation and statistical analyses

To achieve the requisite statistical power, the experiments will include a sufficient albeit not excessive number of animals, adhering to ethical guidelines. Required sample sizes have been calculated with the ClinCalc online tool. Equal enrollment between groups will be achieved, and a power of 0.80, type I error of 0.05 and type II error of 0.2 is aimed for. The study contains the predefined primary endpoints of enhanced survival and energy metabolism. We propose study success not to require the primary objectives to be met. Irrespective of a functional benefit, the secondary outcomes of improved transcriptomic and proteomic characteristics will be investigated. To prevent inflating type I errors, the multiple outcomes will be adjusted for multiplicity using a fallback procedure. The objectives will be tested in the recorded order and, if one objective fails, subsequent tests will be run at a fractionally reduced alpha level, but testing for all three will go ahead. For the primary outcome of survival, three doses of taurine (1-1.5-2mM) will be tested in populations that contain three different genotypes. As an untreated control group is included, this results in a total of 12 independent groups. Based upon previous studies, an incidence of 40 vs. 60% with dichotomous primary endpoint (life or death) is expected, calculating a sample size of 97 per independent group, which will be rounded up to 100 fish per group as a precaution toward possible adverse events. In view of the recessive Mendelian genetics of the *dmd* defect, survival assays for each dose would thus require 400 animals. For the continuous secondary endpoints, transcriptomic and proteomic analyses will be done. Power calculation was based upon published studies and our own preliminary data investigating the mTOR biomarker, and calculated to require sample sizes of 33 (average 45% decrease of mRNA) and 74 (average 30% decrease of protein) per group.

Statistical analysis will be carried out using the GraphPad Prism software package. Outliers will be identified with the Robust regression and Outlier Removal (ROUT) algorithm and excluded from the analysis. Survival analysis will be evaluated using Kaplan-Meier survival curves, and differences between the treatment and control groups will be compared using the log-rank test. The Shapiro-Wilk test will determine if values display normal distribution, informing an appropriate choice of *T*-test, one-way ANOVA or Kruskal-Wallis test and *post-hoc* Tukey's. Bonferroni multiple testing correction will be performed to adjust statistical significance thresholds to prevent false positives. 95% confidence intervals will be reported alongside effect size as Cohen's d representing differences between untreated and treated groups. Differences in means will be analyzed with a Dunn test with Benjamini–Hochberg correction. Levene's testing will evaluate homogeneity of variances. When *p*-values below 0.05 are found, a statistical difference will be assumed.

## 3 Preliminary results from pilot experiments

Feasibility of the study is assured, as the taurine dose and all methodologies have been optimized and tested.

### 3.1 Phenotyping/genotyping

**Figure 1:**
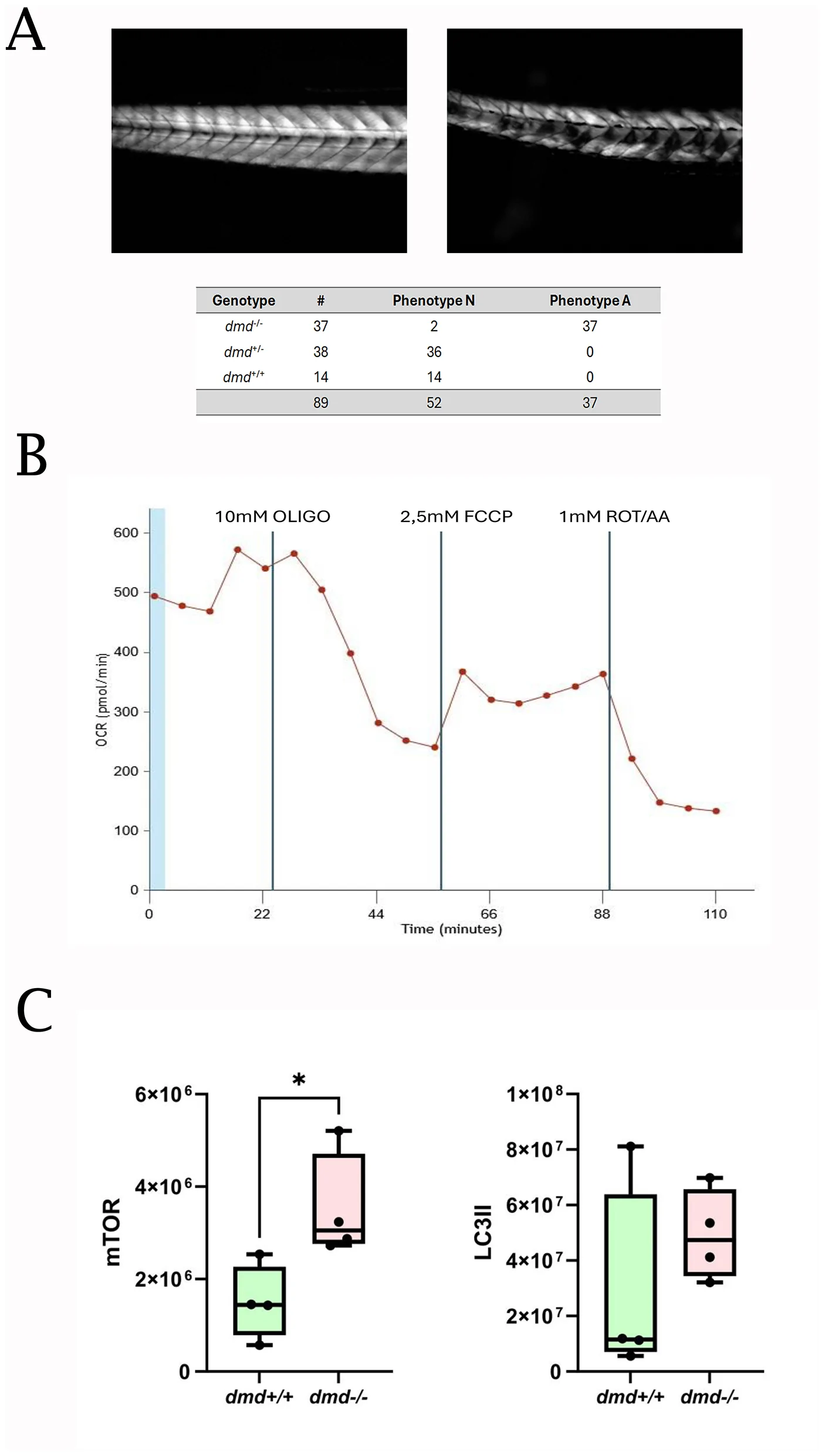
**(A)** Birefringence microscopic images are shown of 4 days post fertilization (dpf) larvae displaying well-organized myotomes classified as normal (N, left) or disorganized myotomes classified as abnormal (A, right) phenotype. **(B)** Representative trajectory of oxygen consumption rate (OCR) obtained with Seahorse Extracellular Flux Analysis in a 5 dpf zebrafish larva after sequential injections. **(C)** Comparative Western blot in samples from 5 dpf zebrafish larvae showed increased mean levels of mechanistic target of rapamycin (mTOR) and lipidated microtubule-associated protein 1A/1B-light chain 3 (LC3II) protein in zebrafish larvae genotyped *dmd*^−/−^, statistically significant for mTOR (unpaired T-test for data with normal distribution, *p* = 0,03*).

We tested birefringence phenotyping of 4 dpf larvae using birefringence microscopy, classifying larvae with bright and well-organized myotomes as normal and larvae with patchy and disorganized myotomes as abnormal (Figure 1A). The phenotype/genotype correlation was tested for a set of 89 4 dpf larvae, appointing the abnormal phenotype with a success rate of 100% for predicting *dmd*^−/−^ genotype.

### 3.2 Seahorse extracellular flux analysis

Conditions for measuring mitochondrial respiratory capacity and glycolysis, represented by OCR and ECAR respectively, were optimized, generating solid trajectories in individual 5 dpf zebrafish larvae (Figure 1B).

### 3.3 RT-qPCR

Primer efficiencies have been tested on serial dilutions of cDNA prepared from genotyped *dmd*^+/−^ zebrafish and calculated from the slope of the standard curve. All primer sets reached acceptable efficiency (E) ranging between 0.80 and 1.20 with the formula E = 10^(−1/slope)^ -1.

### 3.4 Western blotting

A large quantity of commercially available antibodies were tested, as most have not been validated for zebrafish materials, focussing specifically on DMD autophagy pathway disturbances. We determined mTOR protein levels were significantly higher in 5 dpf *dmd*^−/−^ compared to *dmd*^+/+^ larvae (Figure 1C). The increase of mean LC3II protein levels did not reach significance due to deviant levels in one of the samples, yet the observed trend makes it worthwhile to also pursue this protein further. These results are fitting with published results of increased autophagic markers observed in early stages of DMD and converse decreases in later stages of the disease (25). Our preliminary data further supports the proposition that autophagy induction contributes to the initial compensatory phase of the disease, which we propose to use as an outcome measure to compare untreated with taurine-treated larvae.

## Data Availability

The raw data supporting the conclusions of this article will be made available by the corresponding author upon request.
